# Supplementary material for: Identification and validation of autophagy-related genes in Hirschsprung’s disease
Source: PeerJ. 2024 Oct 30;12:e18376. doi: 10.7717/peerj.18376 (PMC11531261; doi:10.7717/peerj.18376)
Supplement: Supplemental Information 1 [file peerj-12-18376-s001.docx]

Supplementary Material

Identification and Validation of Autophagy-Related Genes in

Hirschsprung’s Disease

Ting Yao^1†^, Zenghui Hao^1†^, Wei Fan^1^, Jinbao Han^1^, Shuyu Wang^2^, Zaiqun Jiang^1^, Yunting Wang^1^

,Xiaoqian Yang and Zhilin Xu^1*^

*** Correspondence:** Zhilin Xu ：xzlin333@163.com

# Supplementary Tables

**Table S1 Clinical data of patients with HSCR**

| Age at surgery Sex Associated anomalies Extent  (months) Aganglionosis | Enterocolitis | | |
| --- | --- | --- | --- |
|  | Preoperative Post laparoscopic-assisted | | |
|  | Pull-through |  |  |
| 1 1 Male - Longsegment  2 14 Male PDA Ligation Rectosigmoid  3 2 Female - Rectosigmoid  4 2 Female - Longsegment  5 4 Male ASD Rectosigmoid  6 6 Male - Rectosigmoid  7 8 Male PDA Ligation Rectosigmoid  8 10 Male Cryptorchidism Rectosigmoid  9 4 Male - Rectosigmoid  10 8 Male - Rectosigmoid | Yes -  - Yes  - - |  |  |
|  | - -  - - |  |  |
|  | - - |  |  |
|  | - - |  |  |
|  | - - |  |  |
|  | - - |  |  |
|  | - Yes |  |  |

**TABLE S2 | Functional and pathway enrichment analyses for module genes（****The top 3 terms were selected based on the adjusted P-value**

**rankings when >3 terms were enriched for a given category）**

| **Term** | **Description** | **Count** | **P-value** | **Adjusted P-value** | **Genes** |
| --- | --- | --- | --- | --- | --- |
| Biological processes |  | | | | |
| GO:0006914 | Autophagy | 9 | 7.24e-10 | 6.91e-07 | *ARSB, BAG3, BNIP3, ITPR1, SIRT1, CLN3, MAP1LC3B, CHMP2B, ATG3* |
| GO:0061919 | Process utilizing autophagic mechanism | 9 | 7.24e-10 | 6.91e-07 | *ARSB, BAG3, BNIP3, ITPR1, SIRT1, CLN3, MAP1LC3B, CHMP2B, ATG3,* |
| GO:0016236 | Macroautophagy | 7 | 1.12e-08 | 7.19e-06 | *BAG3, BNIP3, SIRT1, CLN3, MAP1LC3B, CHMP2B, ATG3* |
| Cellular component |  | | | | |
| [GO:0005783](http://amigo.geneontology.org/amigo/term/GO:0005783) | Endoplasmic reticulum | 9 | 4.12e-05 | 0.00278 | *CAPN2, ARSB, EIF2AK3, BNIP3, SERPINA1, ITPR1, EGFR, CLN3, ATF6* |
| GO:0005635 | Nuclear envelope | 5 | 6.58e-05 | 0.00278 | *BNIP3, ITPR1, EGFR, SIRT1, ATF6* |
| [GO:0043231](http://amigo.geneontology.org/amigo/term/GO:0043231) | Bounding membrane of organelle | 9 | 8.49e-05 | 0.00278 | *BNIP3, SERPINA1, GOPC, ITPR1, EGFR, CLN3, MAP1LC3B, CHMP2B, ATF6* |
| Molecular functions |  | | | | |
| [GO:0019899](http://amigo.geneontology.org/amigo/term/GO:0019899) | Enzyme binding | 11 | 2.67e-06 | 0.000513 | *NRG1, CAPN2, EIF2AK3, BNIP3, SERPINA1, EGFR, SIRT1, MAP1LC3B, CDKN2A, ATF6, ATG3* |
| GO:0050839 | Cell adhesion molecule binding | 5 | 0.000134 | 0.01290 | *NRG1, BAG3, ITGA6, EGFR, CHMP2B* |
| GO:0045296 | Cadherin binding | 4 | 0.000357 | 0.0228 | *BAG3, ITGA6, EGFR, CHMP2B* |
| KEGG pathway |  | | | | |
| hsa04140 | Autophagy – animal | 4 | 0.000172 | 0.014603 | *EIF2AK3, BNIP3, ITPR1, ATG3* |
| hsa04218 | Cellular senescence | 4 | 0.000311 | 0.014603 | *CAPN2, ITPR1, SIRT1, CDKN2A* |
| hsa04068 | FoxO signaling pathway | 3 | 0.002543 | 0.044057 | *BNIP3, EGFR, SIRT1* |
