# Supplementary material for: Identification and validation of autophagy-related genes in Hirschsprung’s disease
Source: PeerJ. 2024 Oct 30;12:e18376. doi: 10.7717/peerj.18376 (PMC11531261; doi:10.7717/peerj.18376)
Supplement: Supplemental Information 3 [file peerj-12-18376-s003.docx]

| **ITEM TO CHECK** | **IMPORTANCE** | **DETAILS** |
| --- | --- | --- |
| ****EXPERIMENTAL DESIGN**** |  |  |
| Definition of experimental and control groups | E | Experimental groups included HSCR patients, and control groups included healthy individuals. Samples were matched for age and gender. （Line104-112） |
| Number within each group | E | Each group consisted of 10 individuals. （Line104-105） |
| Assay carried out by core lab or investigator's lab? | D | The assays were carried out in the investigator's lab. |
| Acknowledgement of authors' contributions | D | Acknowledgement of authors' contributions is provided in the document. |
| ****SAMPLE**** |  |  |
| Description | E | Colon tissues from HSCR patients and healthy controls. （Line114-116） |
| Volume/mass of sample processed | D | Approximately 100 mg of colon tissue was processed for each sample. |
| Microdissection or macrodissection | D | Microdissection was used to isolate specific areas of interest from the colon samples. |
| Processing procedure | D | Samples were processed using standard RNA extraction protocols involving Trizol reagent. （Line114-119） |
| If frozen - how and how quickly? | E | Samples were rapidly frozen in liquid nitrogen and stored at -80°C immediately after collection. （Line114-119） |
| If fixed - with what, how quickly? | D | Samples were fixed using formalin immediately after collection and stored in paraffin blocks. |
| Sample storage conditions and duration (especially for FFPE) | D | Frozen samples were stored at -80°C, while FFPE samples were kept at room temperature. Storage duration was up to 2 years. |
| ****NUCLEIC ACID EXTRACTION**** |  |  |
| Procedure and/or instrumentation | E | RNA extraction was performed using Trizol reagent following the manufacturer’s protocol. （Line114-119） |
| Name of kit and details of any modifications | D | The One Step SYBR® PrimeScript™ RT-PCR Kit II was used without modifications. |
| Source of additional reagents used | D | Additional reagents were sourced from Invitrogen and TaKaRa Biotechnology. |
| Details of DNase or RNAse treatment | E | DNase treatment was performed to remove genomic DNA contamination. |
| Contamination assessment (DNA or RNA) | D | Contamination was assessed using no-RT controls in qPCR. |
| Nucleic acid quantification | D | Quantification was performed using a NanoDrop spectrophotometer. |
| Instrument and method | D | RNA quantity and purity were measured using a NanoDrop spectrophotometer. |
| Purity (A260/A280) | D | RNA purity was assessed by measuring the A260/A280 ratio, aiming for values between 1.8 and 2.0. |
| Yield | D | RNA yield was typically around 20-50 µg per 100 mg of tissue. |
| RNA integrity method/instrument | D | RNA integrity was assessed using an Agilent 2100 Bioanalyzer. |
| RIN/RQI or Cq of 3' and 5' transcripts | D | RNA Integrity Number (RIN) values were above 7.0. （Line120-127） |
| Electrophoresis traces | D | Electrophoresis traces were included in the supplementary materials. |
| Inhibition testing (Cq dilutions, spike or other) | E | Inhibition was tested by spiking known quantities of RNA and comparing Cq values. |
| ****REVERSE TRANSCRIPTION**** |  |  |
| Complete reaction conditions | E | Reverse transcription was performed using the One Step SYBR® PrimeScript™ RT-PCR Kit II, with specific conditions detailed below. |
| Amount of RNA and reaction volume | E | 1 µg of RNA was used in a 20 µL reaction volume. |
| Priming oligonucleotide (if using GSP) and concentration | D | Random hexamers were used at a concentration of 50 µM. |
| Reverse transcriptase and concentration | D | PrimeScript™ RT enzyme was used at the manufacturer-recommended concentration. |
| Temperature and time | D | The reverse transcription reaction was carried out at 37°C for 15 minutes, followed by 85°C for 5 seconds. |
| Manufacturer of reagents and catalogue numbers | D | Reagents were obtained from TaKaRa Biotechnology, catalog number RR037A. |
| Cqs with and without RT | E | Cq values were compared between RT and no-RT controls to confirm the absence of genomic DNA contamination. |
| Storage conditions of cDNA | D | cDNA was stored at -20°C until further use. |
| ****qPCR TARGET INFORMATION**** |  |  |
| If multiplex, efficiency and LOD of each assay | E | Multiplex assays were not performed in this study. |
| Sequence accession number | E | Sequence accession numbers for target genes were provided in the supplementary materials. |
| Location of amplicon | D | Amplicons were located within exons to avoid amplification of genomic DNA. |
| Amplicon length | D | Amplicon lengths ranged from 100 to 200 bp. |
| In silico specificity screen (BLAST, etc) | E | Primers were designed using Primer-BLAST to ensure specificity. |
| Pseudogenes, retropseudogenes or other homologs? | D | Potential pseudogenes and homologs were checked using BLAST analysis. |
| Sequence alignment | E | Primer sequences were aligned to the reference genome to ensure specificity. |
| Secondary structure analysis of amplicon | D | Secondary structures were analyzed using mFold. |
| Location of each primer by exon or intron (if applicable) | D | Primers were designed to span exon-exon junctions to avoid amplification of genomic DNA. |
| What splice variants are targeted? | E | Specific splice variants were targeted based on known transcript variants. |
| ****qPCR OLIGONUCLEOTIDES**** |  |  |
| Primer sequences | E | Primer sequences were: EGFR (forward: CCCACTCATGCTCTACAACCC, reverse: TCGCACTTCTTACACTTGCGG); SIRT1 (forward: TGTGTCATAGGTTAGGTGGTGA, reverse: AGCCAATTCTTTTTGTGTTCGTG); CDKN2A (forward: GGGTTTTCGTGGTTCACATCC, reverse: CTAGACGCTGGCTCCTCAGTA); ATG3 (forward: GACCCCGGTCCTCAAGGAA, reverse: TGTAGCCCATTGCCATGTTGG). （Line120-127） |
| RTPrimerDB Identification Number | D | Not applicable for this study. |
| Probe sequences | D | Probe sequences were not used as SYBR Green chemistry was employed. |
| Location and identity of any modifications | D | No modifications were used. |
| Manufacturer of oligonucleotides | E | Primers were synthesized by Invitrogen. |
| Purification method | D | Primers were purified using standard desalting methods. |
| ****qPCR PROTOCOL**** |  |  |
| Complete reaction conditions | E | Reactions were carried out in a final volume of 20 µL, containing 10 µL of SYBR Green Master Mix, 1 µL of each primer (10 µM), 1 µL of cDNA, and 7 µL of nuclease-free water. |
| Reaction volume and amount of cDNA/DNA | E | 1 µL of cDNA was used in a 20 µL reaction volume. |
| Primer, (probe), Mg++ and dNTP concentrations | D | Primer, (probe), Mg |
| **ITEM TO CHECK** | **IMPORTANCE** | **DETAILS** |
| Primer, (probe), Mg++ and dNTP concentrations | D | Primer concentration was 10 µM, Mg++ concentration was 3 mM, and dNTP concentration was 200 µM each. |
| Polymerase identity and concentration | D | Taq polymerase was used at a concentration of 1.25 U per 20 µL reaction. |
| Buffer/kit identity and manufacturer | D | SYBR® Green PCR Master Mix from TaKaRa Biotechnology was used. |
| Exact chemical constitution of the buffer | E | The buffer composition included Tris-HCl, KCl, and MgCl2 at proprietary concentrations. |
| Additives (SYBR Green I, DMSO, etc) | D | SYBR Green I was included in the Master Mix. |
| Manufacturer of plates/tubes and catalog number | D | PCR plates and tubes were obtained from Bio-Rad, catalog number HSP9601. |
| Complete thermocycling parameters | E | The PCR cycling conditions were: initial denaturation at 95°C for 3 minutes, followed by 40 cycles of 95°C for 15 seconds and 60°C for 60 seconds. |
| Reaction setup (manual/robotic) | D | Reaction setup was performed manually using pipettes. |
| Manufacturer of qPCR instrument | E | qPCR was performed on a Bio-Rad CFX96 Real-Time PCR Detection System. |
| ****qPCR VALIDATION**** |  |  |
| Evidence of optimisation (from gradients) | E | Primer optimization was performed using gradient PCR to determine the optimal annealing temperature. |
| Specificity (gel, sequence, melt, or digest) | E | Specificity was confirmed by melt curve analysis and gel electrophoresis of PCR products. |
| For SYBR Green I, Cq of the NTC | D | The Cq value of the non-template control (NTC) was greater than 35, indicating no contamination. |
| Standard curves with slope and y-intercept | E | Standard curves were generated with a slope of -3.32 and y-intercept of 25. |
| PCR efficiency calculated from slope | E | PCR efficiency was calculated to be 100% based on the standard curve slope. |
| Confidence interval for PCR efficiency or standard error | D | The standard error of the PCR efficiency was ±2%. |
| r2 of standard curve | D | The r2 value of the standard curve was 0.998, indicating high linearity. |
| Linear dynamic range | D | The linear dynamic range of the assay spanned 7 orders of magnitude. |
| Cq variation at lower limit | D | The Cq variation at the lower limit of detection was less than 1 cycle. |
| Confidence intervals throughout range | D | Confidence intervals were calculated for all data points within the linear range. |
| Evidence for limit of detection | E | The limit of detection was determined to be 10 copies per reaction. |
| If multiplex, efficiency and LOD of each assay | E | Multiplex assays were not performed in this study. |
| ****DATA ANALYSIS**** |  |  |
| qPCR analysis program (source, version) | D | qPCR analysis was performed using Bio-Rad CFX Manager software, version 3.1. |
| Cq method determination | E | Cq values were determined using the threshold cycle method. |
| Outlier identification and disposition | D | Outliers were identified using Grubbs' test and excluded from analysis. |
| Results of NTCs | E | NTC results confirmed no contamination throughout the experiments. |
| Justification of number and choice of reference genes | D | Reference genes were selected based on their stable expression across samples, with at least three reference genes used per analysis. |
| Description of normalization method | E | Normalization was performed using the geometric mean of multiple reference genes. |
| Number and concordance of biological replicates | D | Each experimental condition was tested in triplicate biological replicates. |
| Number and stage (RT or qPCR) of technical replicates | E | Each sample was tested in duplicate technical replicates at both RT and qPCR stages. |
| Repeatability (intra-assay variation) | E | Intra-assay variation (repeatability) was determined to be less than 2%. |
| Reproducibility (inter-assay variation, %CV) | D | Inter-assay variation (reproducibility) was determined to be less than 5%. |
| Power analysis | E | Power analysis was conducted to ensure adequate sample size, with a power of 0.8 to detect significant differences. |
| Statistical methods for result significance | E | Statistical significance was determined using ANOVA followed by post-hoc Tukey's test. |
| Software (source, version) | D | Data analysis was performed using GraphPad Prism software, version 8.0. |
| Cq or raw data submission using RDML | E | Raw Cq data were submitted in RDML format for transparency and reproducibility. |
