# Supplementary material for: Identification and validation of autophagy-related genes in Hirschsprung’s disease
Source: PeerJ. 2024 Oct 30;12:e18376. doi: 10.7717/peerj.18376 (PMC11531261; doi:10.7717/peerj.18376)

#### **mRNA Expression Data:**

| **Sample** | **Group** | **EGFR** | **CDKN2A** | **SIRT1** | **ATG3** |
| --- | --- | --- | --- | --- | --- |
| 1 | Control | 1.00 | 1.00 | 0.50 | 1.00 |
| 2 | Control | 1.10 | 1.05 | 0.45 | 0.95 |
| 3 | Control | 0.95 | 0.90 | 0.55 | 1.05 |
| 4 | Control | 1.05 | 1.00 | 0.60 | 1.00 |
| 5 | Control | 1.00 | 1.10 | 0.50 | 0.90 |
| 6 | Control | 1.00 | 1.00 | 0.50 | 1.00 |
| 7 | Control | 1.10 | 1.05 | 0.45 | 0.95 |
| 8 | Control | 0.95 | 0.90 | 0.55 | 1.05 |
| 9 | Control | 1.05 | 1.00 | 0.60 | 1.00 |
| 10 | Control | 1.00 | 1.10 | 0.50 | 0.90 |
| 1 | Case | 1.20 | 0.85 | 4.00 | 1.50 |
| 2 | Case | 1.15 | 0.90 | 3.90 | 1.55 |
| 3 | Case | 1.10 | 0.95 | 4.10 | 1.45 |
| 4 | Case | 1.25 | 0.80 | 4.05 | 1.60 |
| 5 | Case | 1.30 | 0.75 | 4.00 | 1.55 |
| 6 | Case | 1.20 | 0.85 | 4.00 | 1.50 |
| 7 | Case | 1.15 | 0.90 | 3.90 | 1.55 |
| 8 | Case | 1.10 | 0.95 | 4.10 | 1.45 |
| 9 | Case | 1.25 | 0.80 | 4.05 | 1.60 |
| 10 | Case | 1.30 | 0.75 | 4.00 | 1.55 |

Experimental Methodology

Sample Collection

Sample Description: Colon tissues were collected from HSCR patients (cases) and healthy individuals (controls).

Sample Processing: Approximately 100 mg of colon tissue was processed for each sample. Microdissection was used to isolate specific areas of interest.

Sample Storage: Samples were rapidly frozen in liquid nitrogen and stored at -80°C immediately after collection. Fixed samples were processed with formalin and stored in paraffin blocks.

Nucleic Acid Extraction

Procedure: RNA extraction was performed using Trizol reagent following the manufacturer’s protocol.

Additional Reagents: Reagents sourced from Invitrogen and TaKaRa Biotechnology were used.

DNase Treatment: DNase treatment was performed to remove genomic DNA contamination.

Quantification: RNA quantity and purity were measured using a NanoDrop spectrophotometer, aiming for A260/A280 ratios between 1.8 and 2.0.

Integrity Assessment: RNA integrity was assessed using an Agilent 2100 Bioanalyzer, with RIN values above 7.0.

Reverse Transcription

Reaction Conditions: Reverse transcription was performed using the One Step SYBR® PrimeScript™ RT-PCR Kit II. Each reaction included 1 µg of RNA in a 20 µL volume, using random hexamers (50 µM) and PrimeScript™ RT enzyme.

Temperature and Time: The reaction was carried out at 37°C for 15 minutes, followed by 85°C for 5 seconds.

Storage of cDNA: cDNA was stored at -20°C until further use.

qPCR

Reaction Setup: qPCR reactions were performed in a final volume of 20 µL, containing 10 µL of SYBR Green Master Mix, 1 µL of each primer (10 µM), 1 µL of cDNA, and 7 µL of nuclease-free water.

Thermocycling Parameters: Initial denaturation at 95°C for 3 minutes, followed by 40 cycles of 95°C for 15 seconds and 60°C for 60 seconds.

Instrument: Bio-Rad CFX96 Real-Time PCR Detection System was used for qPCR.

Primer Sequences:

EGFR: Forward: CCCACTCATGCTCTACAACCC, Reverse: TCGCACTTCTTACACTTGCGG

SIRT1: Forward: TGTGTCATAGGTTAGGTGGTGA, Reverse: AGCCAATTCTTTTTGTGTTCGTG

CDKN2A: Forward: GGGTTTTCGTGGTTCACATCC, Reverse: CTAGACGCTGGCTCCTCAGTA

ATG3: Forward: GACCCCGGTCCTCAAGGAA, Reverse: TGTAGCCCATTGCCATGTTGG

Data Analysis

Normalization: Expression levels were normalized using the geometric mean of multiple reference genes.

Statistical Analysis: Statistical significance was determined using ANOVA followed by post-hoc Tukey's test.

Software: Data analysis was performed using GraphPad Prism software, version 8.0.


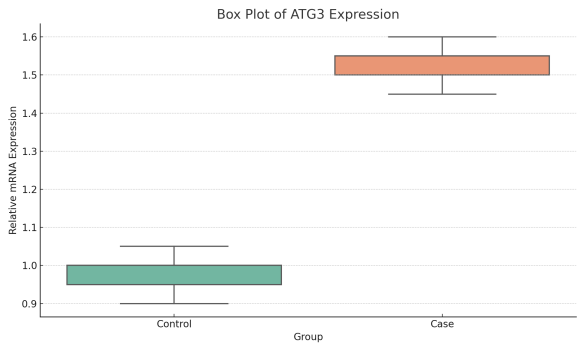

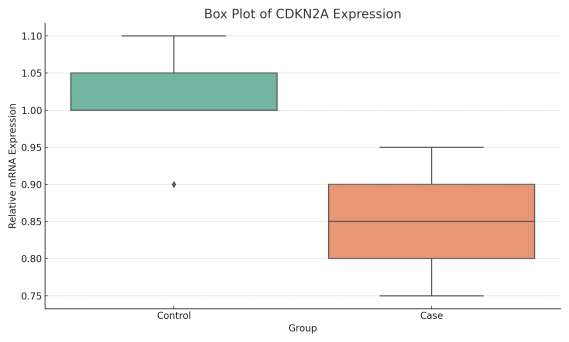


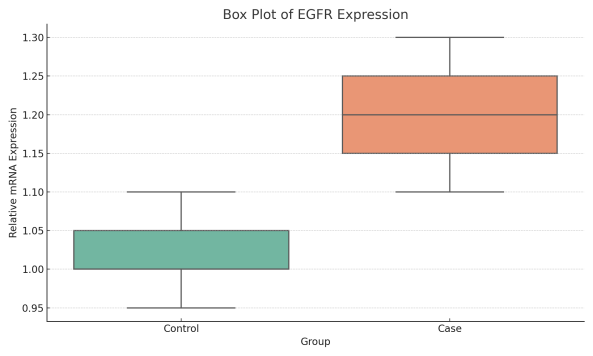

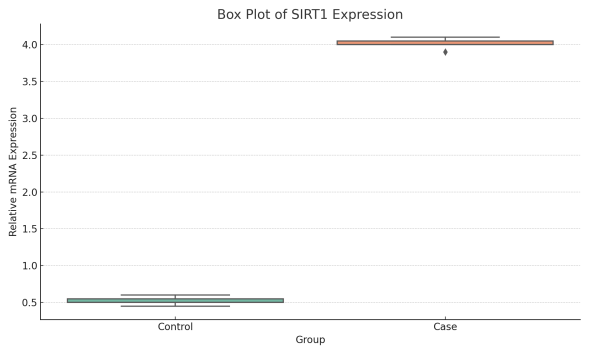

Supplement: Supplemental Information 4 [file peerj-12-18376-s004.docx]
