# Supplementary material for: Identification and validation of autophagy-related genes in Hirschsprung’s disease
Source: PeerJ. 2024 Oct 30;12:e18376. doi: 10.7717/peerj.18376 (PMC11531261; doi:10.7717/peerj.18376)
Supplement: Supplemental Information 5 — The code used for generating the figures and conducting the bioinformatics analysis [file peerj-12-18376-s005.docx]

**PCA Plot (Fig2. Panel A)**

# Load necessary libraries

library(ggplot2)

library(ggfortify)

# Assuming 'data' is your dataset and 'group' is a factor column with "Control" and "Case"

pca_res <- prcomp(data, scale. = TRUE)

# Plot PCA

ggplot() +

geom_point(aes(x = pca_res$x[,1], y = pca_res$x[,2], color = group), size = 3) +

stat_ellipse(aes(x = pca_res$x[,1], y = pca_res$x[,2], color = group), level = 0.95) +

labs(x = paste("PC1 (", round(summary(pca_res)$importance[2,1] * 100, 2), "%)", sep=""),

y = paste("PC2 (", round(summary(pca_res)$importance[2,2] * 100, 2), "%)", sep=""),

title = "PCA Plot") +

theme_minimal()

**Volcano Plot (Fig2.Panel B)**

# Load necessary libraries

library(ggplot2)

# Assuming 'results' is a dataframe with 'log2FoldChange' and 'pvalue'

results$significant <- "Not Significant"

results$significant[results$pvalue < 0.05 & abs(results$log2FoldChange) > 1] <- "Significant"

ggplot(results, aes(x = log2FoldChange, y = -log10(pvalue), color = significant)) +

geom_point(alpha = 0.6, size = 2) +

geom_text(aes(label = ifelse(significant == "Significant", as.character(gene), '')),

vjust = 1.5, hjust = 1.5, check_overlap = TRUE) +

geom_vline(xintercept = c(-1, 1), linetype = "dashed") +

geom_hline(yintercept = -log10(0.05), linetype = "dashed") +

scale_color_manual(values = c("grey", "red", "blue")) +

theme_minimal() +

labs(x = "log2(Fold Change)", y = "-log10(p-value)", title = "Volcano Plot")

**Heatmap (Fig2.Panel C)**

# Load necessary libraries

library(pheatmap)

# Assuming 'data_matrix' is the data matrix with rows as genes and columns as samples

# 'annotation' is a dataframe indicating the sample groups

# Plot heatmap

pheatmap(data_matrix,

annotation_col = annotation,

scale = "row",

clustering_method = "complete",

show_colnames = TRUE,

show_rownames = TRUE,

color = colorRampPalette(c("blue", "white", "red"))(50))

**Violin Plot with Multiple Genes (Fig3，Fig8)**

# Load necessary libraries

library(ggplot2)

# Assuming 'data' is a dataframe with columns: 'Gene', 'Expression', and 'Group'

# 'Gene' is a factor of gene names, 'Expression' is the expression level, and 'Group' is the factor indicating 'Case' or 'Control'

# Panel A and Panel B can be created similarly, with different subsets of 'Gene'

# Example for Panel A

ggplot(data, aes(x = Gene, y = Expression, fill = Group)) +

geom_violin(trim = FALSE) +

**Correlation Heatmap with Significance (Fig4.Panel A)**

# Load necessary libraries

library(corrplot)

# Assuming 'cor_matrix' is the correlation matrix, and 'p_matrix' is the matrix of p-values

# Create a function to get the significance stars

cor.mtest <- function(mat, ...) {

mat <- as.matrix(mat)

n <- ncol(mat)

p.mat <- matrix(NA, n, n)

diag(p.mat) <- 0

for (i in 1:(n - 1)) {

for (j in (i + 1):n) {

tmp <- cor.test(mat[, i], mat[, j], ...)

p.mat[i, j] <- p.mat[j, i] <- tmp$p.value

}

}

colnames(p.mat) <- rownames(p.mat) <- colnames(mat)

p.mat

}

# Calculate the p-values matrix if not available

p_matrix <- cor.mtest(cor_matrix)

# Plot

corrplot(cor_matrix, method = "circle", type = "lower",

p.mat = p_matrix, sig.level = 0.05, insig = "blank",

tl.col = "black", tl.srt = 45,

col = colorRampPalette(c("blue", "white", "red"))(200))

geom_boxplot(width = 0.1, position = position_dodge(0.9), color = "black", outlier.shape = NA) +

stat_summary(fun = median, geom = "point", shape = 18, size = 2, color = "black", position = position_dodge(0.9)) +

scale_fill_manual(values = c("red", "blue")) +

labs(x = "", y = "Gene Expression", title = "Panel A") +

theme_minimal() +

theme(axis.text.x = element_text(angle = 45, hjust = 1))

# Example for Panel B

ggplot(data_b, aes(x = Gene, y = Expression, fill = Group)) +

geom_violin(trim = FALSE) +

geom_boxplot(width = 0.1, position = position_dodge(0.9), color = "black", outlier.shape = NA) +

stat_summary(fun = median, geom = "point", shape = 18, size = 2, color = "black", position = position_dodge(0.9)) +

scale_fill_manual(values = c("red", "blue")) +

labs(x = "", y = "Gene Expression", title = "Panel B") +

theme_minimal() +

theme(axis.text.x = element_text(angle = 45, hjust = 1))

**Detailed Correlation Matrix with Coefficients (Fig4.Panel B)**

# Load necessary libraries

library(ggcorrplot)

# Assuming 'cor_matrix' is the correlation matrix and 'p_matrix' contains the p-values

# Convert p-values to significance stars

stars <- ifelse(p_matrix < 0.001, "***",

ifelse(p_matrix < 0.01, "**",

ifelse(p_matrix < 0.05, "*", "")))

# Combine the correlation coefficients and significance stars

cor_labels <- round(cor_matrix, 2)

cor_labels <- paste0(cor_labels, stars)

# Plot

ggcorrplot(cor_matrix,

method = "square",

type = "lower",

lab = TRUE,

lab_col = "black",

lab_size = 3,

show.legend = TRUE,

title = "Correlation Matrix",

legend.title = "Correlation Coefficient",

ggtheme = theme_minimal(),

colors = c("blue", "white", "red"))

**Enrichment Dot Plot (Fig5. Panel A and B)**

# Load necessary libraries

library(ggplot2)

library(clusterProfiler)

library(enrichplot)

# Assuming 'enrichment_result' is an enrichment result object from clusterProfiler (e.g., from enrichGO or enrichKEGG)

# Panel A

dotplot(enrichment_result, showCategory = 20, split = "ONTOLOGY",

color = "p.adjust", size = "Count") +

scale_color_gradient(low = "blue", high = "red") +

theme_minimal() +

labs(title = "Enrichment Dot Plot (Panel A)")

# Panel B (Assuming you want to split by different categories like BP, CC, MF)

dotplot(enrichment_result, showCategory = 10, split = "ONTOLOGY",

color = "p.adjust", size = "Count") +

scale_color_gradient(low = "blue", high = "red") +

facet_grid(ONTOLOGY ~ ., scales = "free") +

theme_minimal() +

labs(title = "Enrichment Dot Plot (Panel B)")

**Enrichment Network Plot (Fig5. Panel C)**

# Load necessary libraries

library(clusterProfiler)

library(enrichplot)

# Assuming 'enrichment_result' is an enrichment result object

# Plot the enrichment network

cnetplot(enrichment_result, showCategory = 10,

foldChange = geneList, # Replace with your gene list with fold changes

node_label = "all") +

theme_minimal() +

labs(title = "Enrichment Network Plot (Panel C)")

**Circular Chord Diagram (Fig6. Panels A and B)**

# Load necessary libraries

library(circlize)

# Example data: Replace this with your actual data

# Assuming `pathways_genes` is a data frame with two columns:

# `Pathway` and `Gene`. Each row indicates a connection between a gene and a pathway.

# Example for Panel A

genes <- c("NRG1", "BAG3", "CAPN2", "ITGA6", "EGFR", "ARSB", "NAF1", "SIRT1", "CHMP2B", "EIF2AK3", "MAP1LC3B", "GOPC", "CLN3", "ATG3", "ATF6")

pathways <- c("Protein processing in endoplasmic reticulum", "Alzheimer disease", "Focal adhesion",

"EGFR tyrosine kinase inhibitor resistance", "ErbB signaling pathway", "Lysosome",

"FoxO signaling pathway", "Apoptosis", "Autophagy - animal", "Hepatitis C",

"Cellular senescence", "Necroptosis", "Glycosaminoglycan degradation",

"Regulation of actin cytoskeleton", "Autophagy - other")

# Prepare the data in a format suitable for circlize

# Example linkages between genes and pathways (fill in with actual data)

df <- data.frame(

from = sample(genes, 30, replace = TRUE),

to = sample(pathways, 30, replace = TRUE)

)

# Assign colors to the pathways

pathway_colors <- rainbow(length(unique(df$to)))

# Plot the chord diagram

chordDiagram(df, grid.col = c(setNames(pathway_colors, unique(df$to))))

# Add legend for pathways

legend("topright", legend = unique(df$to), fill = pathway_colors, title = "KEGG Pathways")

# Repeat the same for Panel B with different data
